# Morphological characteristics of coronoid process and revisiting definition of coronoid hyperplasia

**DOI:** 10.1038/s41598-023-46289-4

**Published:** 2023-11-29

**Authors:** Sohaib Shujaat, Constantinus Politis, Tom Van Den Bogaert, Pieter Vueghs, Maximiliaan Smeets, Pieter-Jan Verhelst, Edouard Grymonprez, Reinhilde Jacobs

**Affiliations:** 1 OMFS IMPATH Research Group, Department of Imaging & Pathology, Faculty of Medicine, KU Leuven & Oral and Maxillofacial Surgery, University Hospitals Leuven, Leuven, Belgium; 2 King Abdullah International Medical Research Center, Department of Maxillofacial Surgery and Diagnostic Sciences, College of Dentistry, King Saud Bin Abdulaziz University for Health Sciences, Ministry of National Guard Health Affairs, Riyadh, Kingdom of Saudi Arabia; 3 Section of Oral Diagnostics and Surgery, Department of Dental Medicine, Division of Oral Diagnostics and Rehabilitation, Karolinska Institutet, Huddinge, Sweden

**Keywords:** Dentistry, Dental anthropology, Dental radiology

## Abstract

The aim of this study was to assess the morphological characteristics of the coronoid process (CP) and define coronoid hyperplasia (CH) using cadaveric mandibles of a Caucasian population. A sample of 151 adult dry cadaveric mandibles (302 CPs) was acquired. Three distances were measured, which included the width, height, and length of CP. The surface area measurements involved area A: above the width distance line; area B: between incisura mandibulae—Alveolar ridge line and width distance line; area C: between distance lines of width and height. Finally, angulations of the CP and gonial angles were identified. Both length and surface area A + B acted as hyperplastic indicators. Based on the selection criteria, a sample of 197 CPs was included. The hooked shape (59%) was most commonly observed. No significant difference existed between left and right sides (p > 0.05). The mean values of length and surface area A + B were 2.2 ± 0.3 cm and 3.3 ± 0.8 cm^2^, and any values above 2.7 cm (n = 5 CPs- 2.5%) and 5.0 cm^2^ (n = 9 CPs- 4.6%) were described as hyperplastic, respectively. The presented data could act as quantitative reference for differentiating between normal and hyperplastic conditions.

## Introduction

The coronoid process (CP) of the mandibular bone is derived from a Greek word, “korone” meaning “like a crown”^1^. It is a thin triangular eminence that continues anteriorly into the ramus and is bounded posteriorly by mandibular incisurae. The lateral surface of CP provides attachment to temporalis and masseter muscles. Even though CP is rarely mentioned when discussing the functionality of the jaw, it plays a vital role in both mastication and mandibular stabilization^2^.

Until now, morphological characteristics of CP have been mainly described for different Asian and Turkish population groups^3–5^. To our knowledge, no study is available presenting such data in a pure Caucasian population. In addition, the main limitations shared by the majority of prior studies are that they either only focus on the different shapes of CP or the geometric findings are primarily based on height without considering the length, width, surface area and angular components^6–14^. To provide a complete picture of a CP, it is important to add all the aforementioned components to the equation when assessing CP geometry^5^.

Furthermore, amongst various mandibular bone deformities, coronoid hyperplasia (CH) has been largely regarded as an underrecognized rare developmental anomaly mainly responsible for mandibular hypomobility. It was first described by Bernhard Von Langenbeck in 1853 and is defined as an abnormally elongated CP composed of histologically healthy bone^15,16^. It occurs mostly in men during the second decade and approximately 80% of the cases have bilateral involvement^17^. The causative factor of CH is still unknown and poorly understood; however, multiple theories have been hypothesized which include temporalis muscle hyperactivity, genetic inheritance, social and dietary influence, pubertal endocrine stimuli, trauma and syndromic factors^18^. Patients having CH often present with progressive mouth opening limitation without any other symptoms. This restricted mandibular movement is due to impingement of the process onto the posterior aspect of zygomatic bone or inner side of the zygomatic arch during mouth opening function^16^. CH is commonly diagnosed radiologically if its height extends ≥ 1 cm above the inferior border of the zygomatic arch^19^. However, it should be noted that it can still be hyperplastic irrespective of the relationship with the zygomatic process and a proper quantitative definition still lacks in literature^20^. It is unknown which CP should be defined as normal or hyperplastic based on different morphological parameters and which of the parameters offer the best possible approach for quantifying hyperplasia^18^. A possible way to make such a distinction and define a hyperplastic condition would be to establish normal values of CP and identify geometric parameters largely deviating from the norm which are not being impacted by different angular dimensions or condylar morphology.

Therefore, the primary aim of the following in vitro study was to investigate the geometric characteristics of the CP and the secondary aim involved establishing a baseline quantitative data suggesting CH using Caucasian population-based cadaveric mandibles.

## Results

From the total sample of 302 CPs, 83 were excluded due to the presence of damaged CPs. In addition, 11 completely edentulous jaws (22 CPs) were also removed from the overall analysis due to their susceptibility to increased bone resorption and muscular pull which can alter the CP’s morphological shape and also lead to false identification of hyperplastic conditions. Finally, a sample of 197 CPs was included. The hooked shape (n = 115, 59%) was most commonly observed followed by round (n = 38, 19%), sharp (n = 27, 14%) and wide (n = 15, 8%). The number of hooked CPs with a wide incisura (n = 67, 58%) was more compared to the small size (n = 48, 42%). Based on the gonial angle, the majority of CPs had a neutral angle (n = 108, 55%), followed by acute (n = 55, 28%) and obtuse (n = 34, 17%).

The inter-observer variability of all measurements showed an excellent ICC for all variables (Table 1). Table 2 represents the descriptive statistics of all recorded variables. Both left and right CPs had approximately similar mean values. Overall, no significant differences existed between either side. In addition, no significant interaction was observed between different categories with all recorded variables of both sides together and individually based on Sidak or Tukey multiple comparison test (p > 0.05).

**Table 1:** Inter-observer intraclass correlation coefficient (ICC) of coronoid process variables.

Variables	ICC	95% confidence interval
Width (cm)	0.9996	0.9992 to 0.9998
Height (cm)	0.9966	0.9935 to 0.9982
Length (cm)	0.9961	0.9927 to 0.9979
Surface area A (cm^2^)	0.9997	0.9994 to 0.9998
Surface area A + B (cm^2^)	0.9995	0.9990 to 0.9997
Surface area C (cm^2^)	0.9992	0.9985 to 0.9996
Angle X (°)	0.9967	0.9938 to 0.9982
Angle Y (°)	0.9987	0.9976 to 0.9993

Surface area A: above coronoid process (CP) width line, surface area B: between incisura mandibulae—alveolar ridge line and width line, surface area C: between CP height and width, angle X: formed by lines placed tangentially to narrowest point of CP with common endpoint lying at highest CP point, angle Y: formed by one line stretched to lowest point of incisura mandibulae and other crossing point where line of width reaches CP anterior part.

**Table 2:** Descriptive statistics of distances, surface areas and angular values of the coronoid process.

Variables	Left side	Right side	Both sides
Width (cm)	2.3 ± 0.3	2.2 ± 0.2	2.3 ± 0.3
Height (cm)	1.7 ± 0.3	1.6 ± 0.3	1.7 ± 0.3
Length (cm)	2.2 ± 0.3	2.2 ± 0.3	2.2 ± 0.3
Surface area A (cm^2^)	1.9 ± 0.5	1.9 ± 0.5	1.9 ± 0.5
Surface area A + B (cm^2^)	3.3 ± 0.8	3.4 ± 0.8	3.3 ± 0.8
Surface area C (cm^2^)	1.9 ± 0.5	1.9 ± 0.5	1.9 ± 0.5
Angle X (°)	48.2 ± 7.9	48.7 ± 7.6	48.4 ± 7.7
Angle Y (°)	68.5 ± 7.7	68.1 ± 7.3	68.2 ± 8.0

Surface area A: above coronoid process (CP) width line, surface area B: between incisura mandibulae—alveolar ridge line and width line, surface area C: between CP height and width, angle X: formed by lines placed tangentially to narrowest point of CP with common endpoint lying at highest CP point, angle Y: formed by one line stretched to lowest point of incisura mandibulae and other crossing point where line of width reaches CP anterior part.

Table 3 describes the mean values of the CPs based on different gonial angles. When examining the relation between the length/surface area A + B and gonial angle no significant differences were detected using Sidak or Tukey multiple comparison test, indicating their applicability for assessing hyperplasia as these parameters were not influenced by the gonial angle. Furthermore, the coronoid angulation variables X and Y were also correlated with length and surface area A + B values using Pearson rank correlation test, to observe whether these angulations impacted the outcome. The findings suggested a negative correlation between these parameters (angle X-length, r = − 0.26; angle Y-length, r = − 0.56; angle X-surface area A + B, r = − 0.17; angle Y- surface area A + B, r = − 0.34), whereas a moderately strong correlation existed between length and surface area A + B (r = 0.79).

**Table 3:** Distribution and significance of the relationship between coronoid process geometric characteristics at different gonial angles.

Variables	Distribution of values based on different gonial angles			Significance of relationship between variables at different gonial angles (p-value)		
	A	B	C	A–B	A–C	B–C
Width (cm)	2.2 ± 0.3	2.3 ± 0.2	2.4 ± 0.2	0.28	**0.01**	**0.02**
Height (cm)	1.6 ± 0.3	1.7 ± 0.3	1.8 ± 0.3	**0.04**	** < 0.01**	**0.02**
Length (cm)	2.1 ± 0.3	2.2 ± 0.3	2.2 ± 0.3	0.45	0.13	0.24
Surface area A (cm^2^)	1.8 ± 0.6	1.8 ± 0.5	1.9 ± 0.5	0.25	0.12	0.42
Surface area A + B (cm^2^)	3.4 ± 0.9	3.3 ± 0.8	3.1 ± 0.6	0.81	0.14	0.13
Surface area C (cm^2^)	1.7 ± 0.5	1.9 ± 0.5	2.2 ± 0.5	0.05	** < 0.01**	**0.01**
Angle X (°)	50.0 ± 9.2	48.3 ± 6.3	45.2 ± 8.4	0.23	**0.01**	**0.01**
Angle Y (°)	70.0 ± 10.2	66.7 ± 7.2	66.6 ± 5.9	0.11	**0.08**	0.38

Gonial angle A (< 120°), B (120–130°), C (> 130°), surface area A: above coronoid process (CP) width line, surface area B: between incisura mandibulae—Alveolar ridge line and width line, surface area C: between CP height and width, angle X: formed by lines placed tangentially to narrowest point of CP with common endpoint lying at highest CP point, angle Y: formed by one line stretched to lowest point of incisura mandibulae and other crossing point where line of width reaches CP anterior part.

*Bold values indicate statistical significance (p < 0.05) with Sidak or Tukey multiple comparison test.

**Figure 1:**
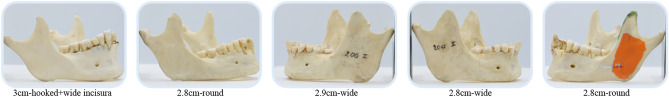
Mandibles with coronoid hyperplasia based on length.

A mean CP length of 2.2 ± 0.3 cm (range: 1.5–3.0 cm) was recorded. Any length above 2.7 cm was described as hyperplastic. The findings suggested a total of 5 [2.5%] of the 197 CPs as hyperplastic (Fig. 1).

**Figure 2:**
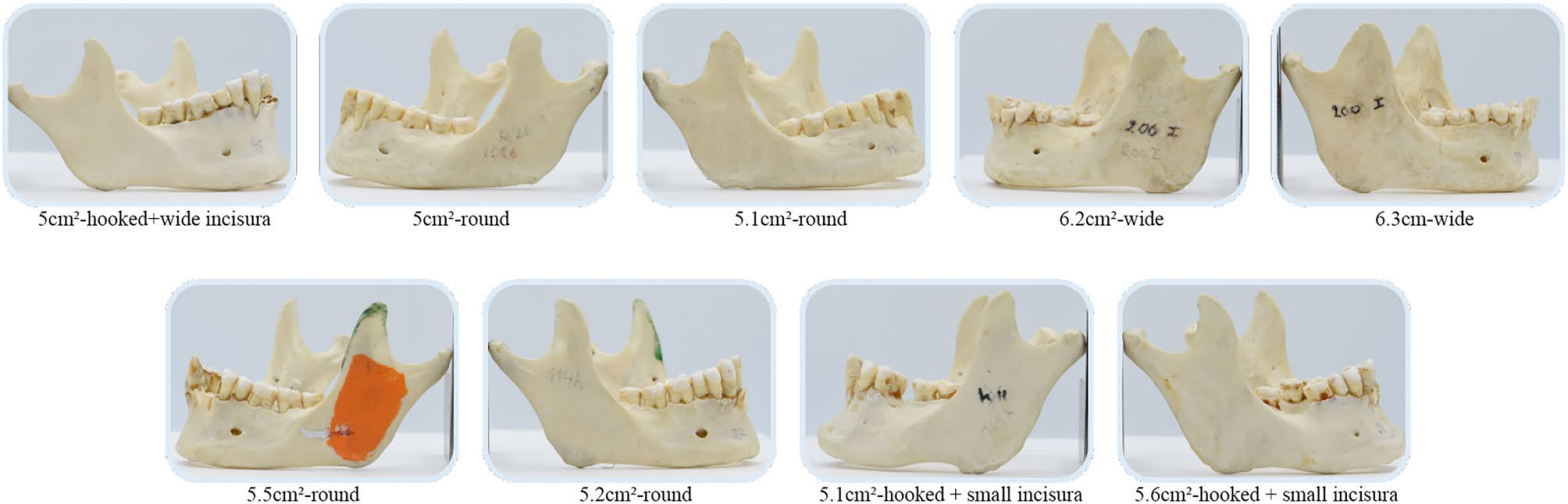
Mandibles with coronoid hyperplasia (CP) based on surface area A + B, where area A: above CP width line, area B: between incisura mandibulae—Alveolar ridge line and width line.

As for the surface area A + B, mean value of 3.3 ± 0.8 cm^2^ (range: 1.8–6.3 cm^2^) was observed with all values above 5.0 cm^2^ being described as hyperplastic. This equated to 9 CPs [4.6%] as hyperplastic (Fig. 2). Amongst these 9 CPs, all the 5 hyperplastic CPs based on length were also included.

## Discussion

The CP acts as a vital structure due to its clinical applicability, as it can be used as a grafting material for the reconstruction of osseous defects such as maxillomandibular fracture, alveolar defects, and sinus augmentation. In addition, the discriminating features of CP can act as an anthropological marker to identify sex, age and gender and race of a person^21^. So far, the morphological characteristics of CP in a Caucasian population group still remains to be investigated and no detailed quantitative definition of CH has been established in literature. Hence, the following study was conducted to quantitatively assess CP and to identify which CPs should be considered hyperplastic in nature based on the deviation of indicators value from the normal mean.

The present study suggested that hooked shape was most commonly observed accounting for 59% of the total number of CPs, which was in accordance with the North Indian, West Indian, Northwestern Turkish and Bangladeshi population groups^11,22–24^. The triangular shape had a higher frequency in Southern Indian and Nepalese population^4,14^, whereas another study conducted in Western Turkish region found round shape to be more prevalent^5^. Most of the previous studies only provided the height of the CP which ranged between 1.39 and 1.53 cm^18^. In contrast, the height of CP which in the present study showed a value of 1.70 cm. This slightly larger value could have been due to the genetic constitution, dietary habits or altered temporalis muscle activity, all of which have been found to influence the shape and size of CP^18^. Hence, there is a need to isolate and study the impact of these influential factors for a better understanding of population-specific differences.

In most of the studied population groups, right CP was generally larger compared to the left side which might be due to muscle hyperactivity and preferential chewing on the right side^5^. On the contrary, our findings suggested no significant difference between both sides which could have been due to the equal force distribution on both CPs without any chewing side preference. Further studies are required to investigate the impact of chewing habits onto the bilateral CP’s size in Caucasian population in an attempt to confirm whether both sides show equal force distribution and muscular activity.

In relation to the CH assessment methodologies, prior studies primarily focused on the height of the CP based on the ratio of CP to the condylar process, where a ratio of approximately > 1.0 has been proposed as the definition CH which might slightly vary depending on the population group being studied^25^. Kubota et al.^26^ used coronoid-gonion and condyle-gonion distances ratio for assessing CH on panoramic images. Travassol et al.^27^ measured the coronoid-condylar ratio using landmarks on computed tomographic images. The diagnosis of CH based on such coronoid-condylar ratios can be misleading as it mainly relies on the condylar geometry and any abnormality within this structure would have a definite negative impact on the accurate identification of hyperplastic conditions^18^. In addition, the radiological diagnosis of CH relies on the height of the CP in relation to zygomatic arch^19^. The present study suggested that the CP height was significantly impacted by the gonial angulation, thereby, identifying CH based on this variable would lead to measurement bias and its application should be avoided when differentiating hyperplastic from normal conditions. Moreover, when examining the relationship between zygoma and CP it should be emphasized that the vertical growth of the CP is not the only variable that lays the foundation of mechanical obstruction between both structures. When passing through zygoma, the horizontal span is another important factor which has been ignored^5^. Hence, the proposed methodology of using length and surface area A + B as hyperplastic indicators could be considered a more effective alternative for assessing CH which is not impacted by the condylar geometry. Although muscle activity was not investigated due to the in vitro design of the study, it has been linked with the gonial angle of the jaw. The evidence suggests that acute angulation could be a secondary symptom of stronger jaw musculature^28,29^. Subsequently it would be logical to assume that there could be a positive correlation between the gonial angle and the size of CP in hyperplastic conditions. However, our findings indicated no significant impact of different categories of gonial angles on either length or surface area A + B, unlike other geometric variables. Hence, further showcasing their usefulness as an indicator for hyperplastic assessment. Nevertheless, it is recommended to perform further radiological studies to confirm if no true correlation exists between these variables.

The process of applying these cadaveric findings to clinical practice could be enhanced through the use of computed tomography (CT)/cone-beam CT imaging datasets of patients through the process of the segmentation^30^, which can enable the generation of three-dimensional virtual model of CP and surrounding structures such as zygomatic arch and condylar bone. This approach could offer a more precise depiction of the CP’s morphology in a patient and assessing its interrelationship with the surrounding anatomical structures. Furthermore, the use of superimposition and color-coded mapping^31^ to demonstrate the three-dimensional surface-based differences in the CPs could enhance our understanding of distinguishing between normal and hyperplastic processes, and identifying which surfaces contribute towards hyperplasia, rather than relying solely on two-dimensional linear measurements. This could guide to identify the average CP and CH in patients. Simultaneously, the application of voxel-based or surface-based superimposition for integration of data from multiple imaging modalities^30^, such as by registering CT/CBCT, magnetic resonance and ultrasonographic imaging datasets could offer an improved methodology for determining the combined influence of skeletal, soft tissue and muscular factors, respectively, which might lead to the development of hyperplastic conditions. Incorporating such techniques could enhance the forensic process by providing a realistic three-dimensional replica of the CP for identification tasks. From a clinical perspective, such a three-dimensional approach could potentially improve the surgical decision-making process. For instance, it could help to determine whether patients with CH could serve as a potential source of bone graft for oral or maxillofacial reconstruction procedures without causing any disturbances. Additionally, it could improve the diagnostic criteria for ascertaining if CH could be a causative factor of restricted jaw mobility or temporomandibular joint disorders.

The strength of the study was the first-time reporting of CP shapes and geometric data in a Caucasian population and defining CH based on hyperplastic indicators. In addition, certain limitations were also observed. Firstly, isolated cadaveric mandibles do not take into account morphology of the zygoma and other variables such as, muscular activity and social and dietary factors, hence it was impossible to map out all variables involved leading to either normal coronoid development or mechanical obstruction due to CH. It is recommended to perform future studies using CT/CBCT and MR imaging datasets for a better understanding of the phenomena and identifying factors which might cause the abnormal elongation of the CP. Secondly, the study only included mandibles of male subjects which might not represent a female population. As CH is predominantly found in males, endocrine stimuli is also one particular factor to take into consideration^18^. Thirdly, the presented definition of CH is based on only Caucasian adult cadavers, which might be applicable to other population groups. Thereby, it is recommended to define CH based on the proposed indicators which overcome the limitations of prior methodologies and also provides a complete picture of the abnormal CP elongation. Finally, coronoid hypoplasia was not defined as no cases were found deviating lower than the norm values. So far, a limited number of cases (less than 10) having hypoplastic CPs have been identified in literature^32^. Thereby, further research is also recommended for quantitatively defining hypoplastic conditions to better understand the underlying causative factors.

In conclusion, the hook-shaped morphological variation of the CP was most commonly observed without any significant difference between left and right side. According to the quantitative hyperplastic indicators, CH was defined as any process having a size and length of more than 5 cm^2^ and 2.7 cm, respectively. As CH often remains undiagnosed, the proposed methodology and cut-off values of a hyperplastic condition on dry cadaver mandibles might support further research for radiologically differentiating between normal and hyperplastic conditions. This would in turn help to identify the factors responsible for this rare condition and improve treatment planning of using CP as a grafting material or modeling morphologically accurate mandible to be used in patients with mandibular defects with better clinical outcomes. Furthermore, the findings of the present study would be of imminent value from an anthropological and forensic point of view where the data could act as a quantitative reference for differentiating CPs between Caucasian and other population groups.

## Methods

This in vitro research was approved by the Ethical Review Board of the Faculty of Medicine, KU Leuven, Leuven, Belgium (registration no.:NH019 2019-09-03). It was conducted in compliance with the World Medical Association Declaration of Helsinki on medical research. All methods were carried out in accordance with relevant guidelines and regulations. The mandibles utilized in this investigation were procured from cadavers, with informed consent obtained from the subjects and/or their legal guardians for the donation of their remains for scientific and educational purposes prior to their death.

### Data acquisition

**Figure 3:**
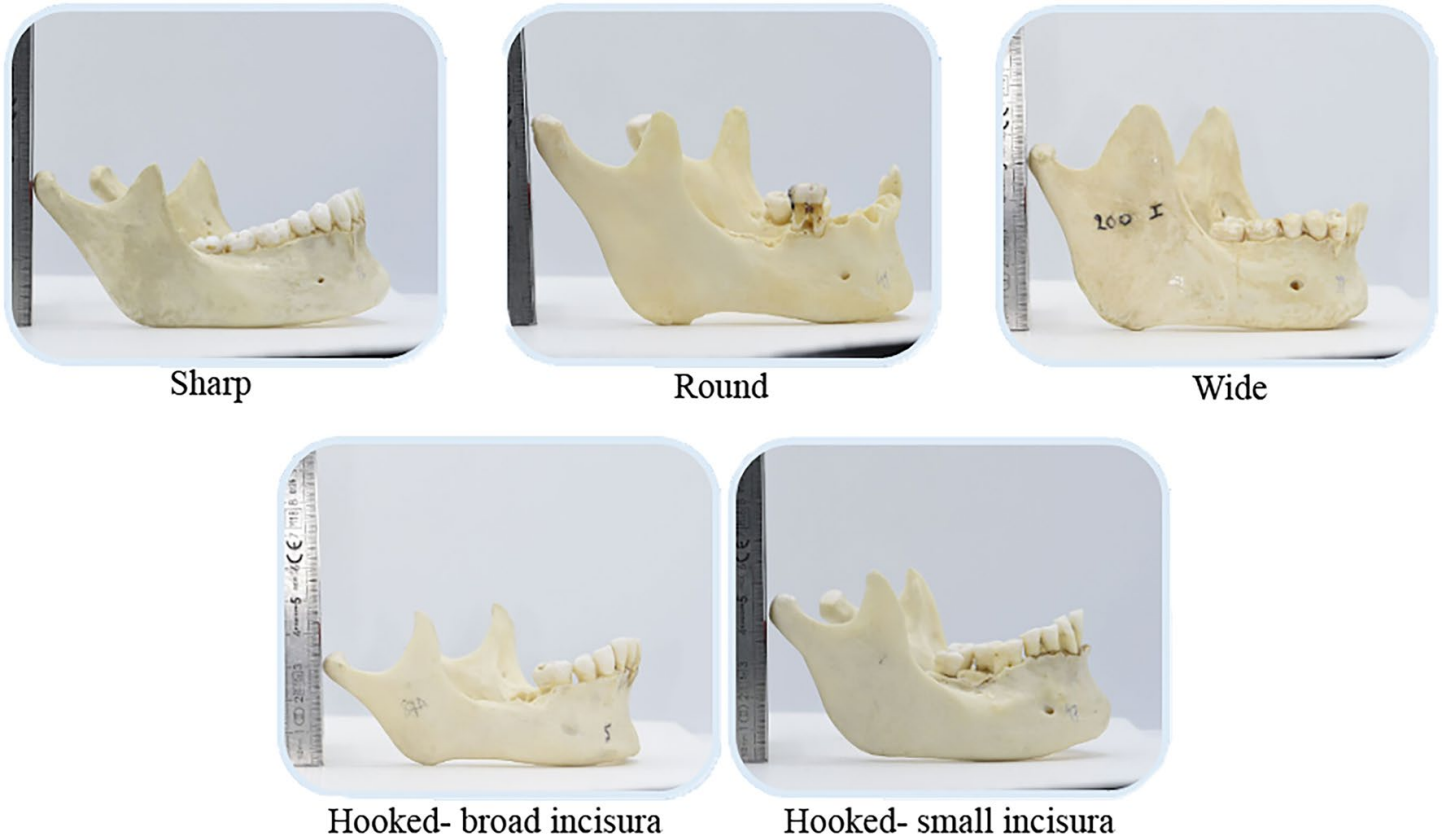
Different shapes of coronoid process.

A total of 151 adult Caucasian dry cadaveric mandibles (302 CPs) of male subjects were acquired. Inclusion criteria consisted of CPs without any skeletal abnormalities. The mandibles with fractured or damaged CPs and edentulous jaws were excluded. Based on the CP shape, it was categorized as either sharp, round, wide or hooked. In addition, the hooked coronoid was further subdivided depending on the depth of incisura as broad or small (Fig. 3).

A Nikon D7000 camera with manual exposure of M250 and focal length of F14 was used to acquire pictures of dry mandibles from both sides. For standardization, all mandibles were placed against a ruler with the mandibular plane parallel to the floor and photographed by placing the camera perpendicular to the ramus. The acquired images were exported to Adobe Photoshop software (Version: 24.1.1, Adobe Systems, San Jose, CA, USA). The number of pixels (px) corresponding to a length of 10 cm were quantified using the ruler positioned next to each mandible. The recorded variables included linear dimensions, surface area and angulation of the CP. Supplementary Figs. S1–S24 provides a detailed description of the complete workflow for calculating the aforementioned variables. The measurements of CP variables were conducted by two observers, where control measurements were carried out for 20% of the data (40 CPs per observer) to assess inter-observer variability.

### Linear dimensions

**Figure 4:**
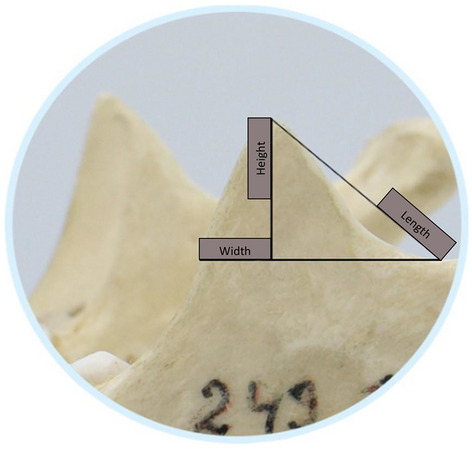
Linear dimensions of coronoid process.

Three distances were measured, which included width, height and length of CP. Firstly, a line was drawn parallel to the mandibular plane starting from the lowest point of the incisura to the anterior part of the CP for calculating the width. Thereafter, the height was determined by measuring the highest point of the CP perpendicular to the line of width. Finally, a line was drawn from the lowest point of the incisura mandibulae to the highest point of the CP for assessing its length (Fig. 4). The resulting output was recorded in centimeters using the ruler tool of the software.

### Surface area

**Figure 5:**
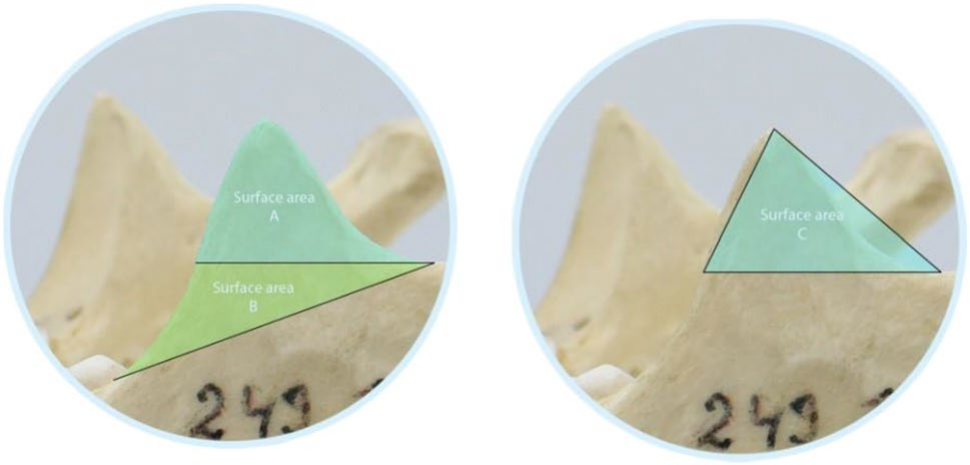
Recorded surface areas of coronoid process (CP), where area A: above CP width line, area B: between incisura mandibulae—Alveolar ridge line and width line, area C = between CP height and width.

The surface area of CP was measured by applying the magnetic lasso tool which made accurate demarcation feasible, and three surface areas were calculated i.e. A, B and C. The surface area A corresponded to the area above the horizontal line of width distance. For surface area B, a line was drawn from the lowest point of the incisura mandibulae to the mandibular alveolar ridge, and it corresponded to the area between this line and the width line. Lastly, surface area C was calculated by defining a triangle based on the CP’s height and width (Fig. 5). The number of px were converted to cm^2^ using the following formula: Px/(Px/10 cm)^2^ × 100.

### Angulations

**Figure 6:**
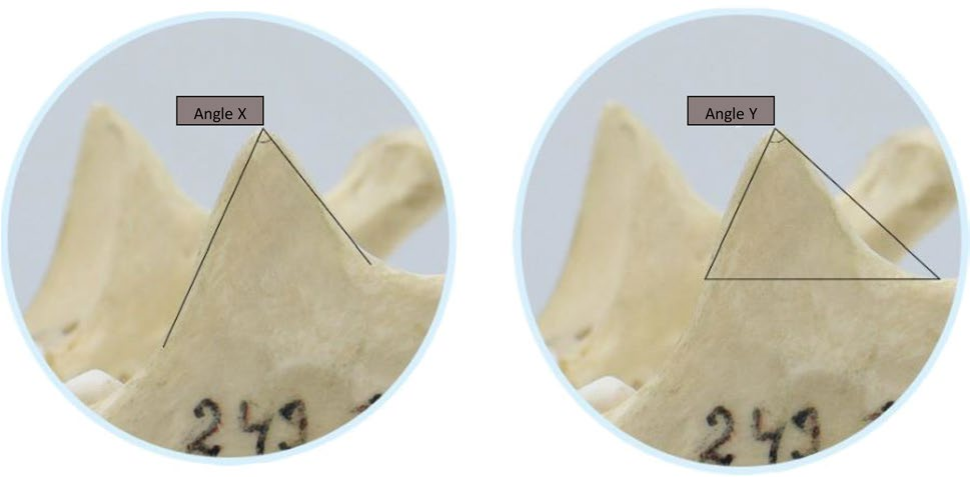
Angular variables (X and Y) of the coronoid process, where angle X: formed by lines placed tangentially to narrowest point of CP with common endpoint lying at highest CP point, angle Y: formed by one line stretched to lowest point of incisura mandibulae and other crossing point where line of width reaches CP anterior part.

Two angles (X and Y) were defined using the ruler tool. Angle X was measured with the common endpoint lying at the highest point of the CP and the lines were placed tangentially to the narrowest point of the CP. To quantify angle Y, one line was stretched to the lowest point of the incisura mandibulae and the other crossed the point where the line of width reached the anterior part of the CP (Fig. 6).

Lastly, the CPs were classified into three distinct groups according to the gonial angle measurements. The angulations were defined as follows: less than 120° (acute), 120–130° (neutral), and greater than 130° (obtuse). Gonial angle was defined as the angle formed by the intersection of ramal and mandibular lines, with the ramal line tangential to the posterior border of the mandible, and the mandibular line to the lower border of the mandible through gnathion.

### Coronoid hyperplasia

The hyperplasia evaluation was performed based on two previously measured indicators: length and surface area A + B, which were referred to as ‘*hyperplastic indicators*’. Based on the indicators, CH was defined as ‘*hyperplastic indicator value differing from the normal mean by two or more standard deviations*.’

Length was chosen as an indicator because it takes into consideration both height and anterior extension of the CP which provides a complete dimensional picture of the CH, unlike width and height which only consider distances in a single plane. On the other hand, surface area A + B acted as the other indicator which avoids measurement bias due to variability of the gonial angle. In contrast, the individual surface areas A and C primarily reflect the superior projection of the CP, which are susceptible to measurement bias for CH assessment due to fluctuations in gonial angulations. Moreover, the relationship between different CP variables (distances, surface areas and angles) and gonial angles was assessed, as such to further confirm whether both length and surface area A + B remained unaffected by gonial angulation and could act as hyperplastic indicators.

### Statistical analysis

Data were analyzed using S-plus for Linux 8.0 (Tibco Software, Palo Alto, CA). Mean and standard deviation values were calculated. Inter-observer was assessed by the intraclass correlation coefficient (ICC) at a 95% confidence interval, where poor ≤ 0.50, moderate = 0.50–0.75, good = 0.75–0.90, and excellent > 0.90^33^. A linear mixed-effects model was applied with CP categories and jaw side as crossed fixed factors and complete mandible as random factor. Residual analysis by means of a normal quantile plot and residual dot plot following exclusion of obvious outliers showed that the basic assumptions of the model were met.

The fixed-effects coefficients of the linear mixed effect model, along with their variance–covariance matrix, were used to conduct comparisons. These comparisons were performed for each side apart and sides were compared for each category apart. The p-values were corrected for simultaneous hypothesis testing according to Sidak^34^. In case no interaction was found, categories were compared for all categories together and a correction for simultaneous hypothesis testing was applied according to Tukey^35^. In addition, the aforementioned analysis was also conducted to assess the differences between gonial angles. The Pearson rank correlation test was conducted to assess the correlation between different variables.

### Supplementary Information

Supplementary Figures.

## Data Availability

The datasets used and/or analysed during the current study available from the corresponding author on reasonable request.

## References

[1] Cascarini L (2007). Mandibular etymologies. Br. Dent. J..

[2] Mezey SE, Müller-Gerbl M, Toranelli M, Türp JC (2022). The human masseter muscle revisited: First description of its coronoid part. Ann. Anat. Anat. Anz..

[3] Hossain SA, Hossain SM, Banna FA (2011). Variations in the shape of the coronoid process in the adult human mandible. Bangladesh J. Anat..

[4] Sahithi D, Reddy S, Teja DD, Koneru J, Praveen KN, Sruthi R (2016). Reveal the concealed–morphological variations of the coronoid process, condyle and sigmoid notch in personal identification. Egypt. J. Forensic Sci..

[5] Keselik GA, Malas MA (2021). Investigation of morphometric parameters of processus coronoideus and mandible in human dry mandibles between sides. Genel Tıp Dergisi.

[6] Isaac B, Holla SJ (2001). Variation in the shape of the coronoid process in the adult human mandible. J. Anat. Soc. India.

[7] Khan TA, Sharieff JH (2011). Observation on morphological features of human mandibles in 200 South Indian subjects. Anat. Karnataka.

[8] Prajapati VP, Malukar O, Nagar SK (2011). Variations in the morphologyical appearance of the coronoid process of human mandible. Int. J. Med. Res..

[9] Nirmale VK, Mane UW, Sukre SB, Diwan CV (2012). Morphological features of human mandible. Int. J. Recent Trends Sci. Technol..

[10] Shakya S, Ongole R, Nagraj SK (2013). Morphology of coronoid process and sigmoid notch in orthopantomograms of South Indian population. World J. Dent..

[11] Tapas S (2014). Morphological variations of coronoid process in dry adult human mandibles. Ind. J. Basic Appl. Med. Res..

[12] Subbaramaiah M, Bajpe R, Jagannatha SR, Jayanthi KS (2015). A study of various forms of mandibular coronoid process in determination of sex. Indian J. Clin. Anat. Physiol..

[13] Suragimath G, Ashwinirani SR, Christopher V, Bijjargi S, Pawar R, Nayak A (2016). Gender determination by radiographic analysis of mental foramen in the Maharashtra population of India. J. Forensic Dent. Sci..

[14] Chaulagain R, Chaudhary S, Poudel P, Gautam A (2022). Morphological variation of coronoid process, sigmoid notch, and condylar process among patients of tertiary care centre of Nepal. J. Kathmandu Med. Coll..

[15] Von Langenbeck B (1861). Angeborene kleinheit der unterkiefer. Langenbeck’s Arch..

[16] Erdem S, Erdem S (2022). Investigation of coronoid process hyperplasia using Levandoski analysis on panoramic radiographs. World J. Radiol..

[17] Wenghoefer M, Merkx M, Steiner M, Götz W, Meijer GJ, Bergg SJ (2006). Hyperplasia of the coronoid process. Asian J. Oral Maxillofac. Surg..

[18] Goh YC, Tan CC, Lim D (2020). Coronoid hyperplasia: A review. J. Stomatol. Oral Maxillofac. Surg..

[19] Galiè M, Consorti G, Tieghi R, Denes SA, Fainardi E, Schmid JL, Neuschl M, Clauser L (2010). Early surgical treatment in unilateral coronoid hyperplasia and facial asymmetry. J. Craniofac. Surg..

[20] Izumi M, Isobe M, Toyama M, Ariji Y, Gotoh M, Naitoh M, Kurita K, Ariji E (2005). Computed tomographic features of bilateral coronoid process hyperplasia with special emphasis on patients without interference between the process and the zygomatic bone. Oral Surg. Oral Med. Oral Pathol. Oral Radiol. Endodontol..

[21] Nayak S, Patra S, Singh G, Mohapatra C, Rath S (2015). Study of the size of the coronoid process of mandible. IOSR J. Dent. Med. Sci..

[22] AkramHossain SM, Moshadeq Hossain SM, Banna FAMH (2011). Variation in the shape of the coronoid process in the adult human mandible. Bangladesh J. Anat..

[23] Bakirci S, Ari I, Kafa IM (2013). Morphometric characteristics and typology of the coronoid process of the mandible. Acta Med. Medterr..

[24] Kadam SD, Roy PP, Ambali MP, Doshi MA (2015). Variation in the shape of coronoid process in dry mandible of Maharastra population. Int. J. Anat. Res..

[25] Stopa Z, Wanyura H, Kowalczyk P (2013). Coronoid-condylar index in assessing of mandibular coronoid hyperplasia. Preliminary results. Adv. Med. Sci..

[26] Kubota Y, Takenoshita Y, Takamori K, Kanamoto M, Shirasuna K (1999). Levandoski panographic analysis in the diagnosis of hyperplasia of the coronoid process. Br. J. Oral Maxillofac. Surg..

[27] Tavassol F, Spalthoff S, Essig H, Bredt M, Gellrich NC, Kokemüller H (2012). Elongated coronoid process: CT-based quantitative analysis of the coronoid process and review of literature. Int. J. Oral Maxillofac. Surg..

[28] Ahila SC, Sasikala C, Kumar BM, Tah R, Abinaya K (2016). Evaluation of the correlation of ramus height, gonial angle, and dental height with different facial forms in individuals with deep bite disorders. Ann. Med. Health Sci. Res..

[29] Amorim MM, Borini CB, de Castro Lopes SL, de Oliveira TD, Bérzin F, Caria PH (2010). Relationship between the angle of the coronoid process of the mandible and the electromyographic activity of the temporal muscle in skeletal class I and III individuals. J. Oral Rehabil..

[30] Shujaat S, Bornstein MM, Price JB, Jacobs R (2021). Integration of imaging modalities in digital dental workflows-possibilities, limitations, and potential future developments. Dentomaxillofac. Radiol..

[31] Hwang HS, Jiang T, Sun L, Lee KM, Oh MH, Biao Y, Oh HK, Bechtold TE (2019). Condylar head remodeling compensating for condylar head displacement by orthognathic surgery. J. Craniomaxillofac. Surg..

[32] Aslan KB, Çakir B (2023). Mandibular coronoid aplasia with condylar hypoplasia: A very rare case with the review of literature. Turk. Klin. J. Dent. Sci..

[33] Koo TK, Li MY (2016). A guideline of selecting and reporting intraclass correlation coefficients for reliability research. J. Chiropr. Med..

[34] Sidak Z (1967). Rectangular confidence regions for the means of multivariate normal distributions. J. Am. Stat. Assoc..

[35] Tukey JW (1949). Comparing individual means in the analysis of variance. Biometrics.

