# Supplementary material for: Morphological characteristics of coronoid process and revisiting definition of coronoid hyperplasia
Source: Sci Rep. 2023 Nov 29;13:21049. doi: 10.1038/s41598-023-46289-4 (PMC10687078; doi:10.1038/s41598-023-46289-4)
Supplement: Supplementary file 1 — Supplementary Figures. [file 41598_2023_46289_MOESM1_ESM.docx]

**Supplementary Figures**


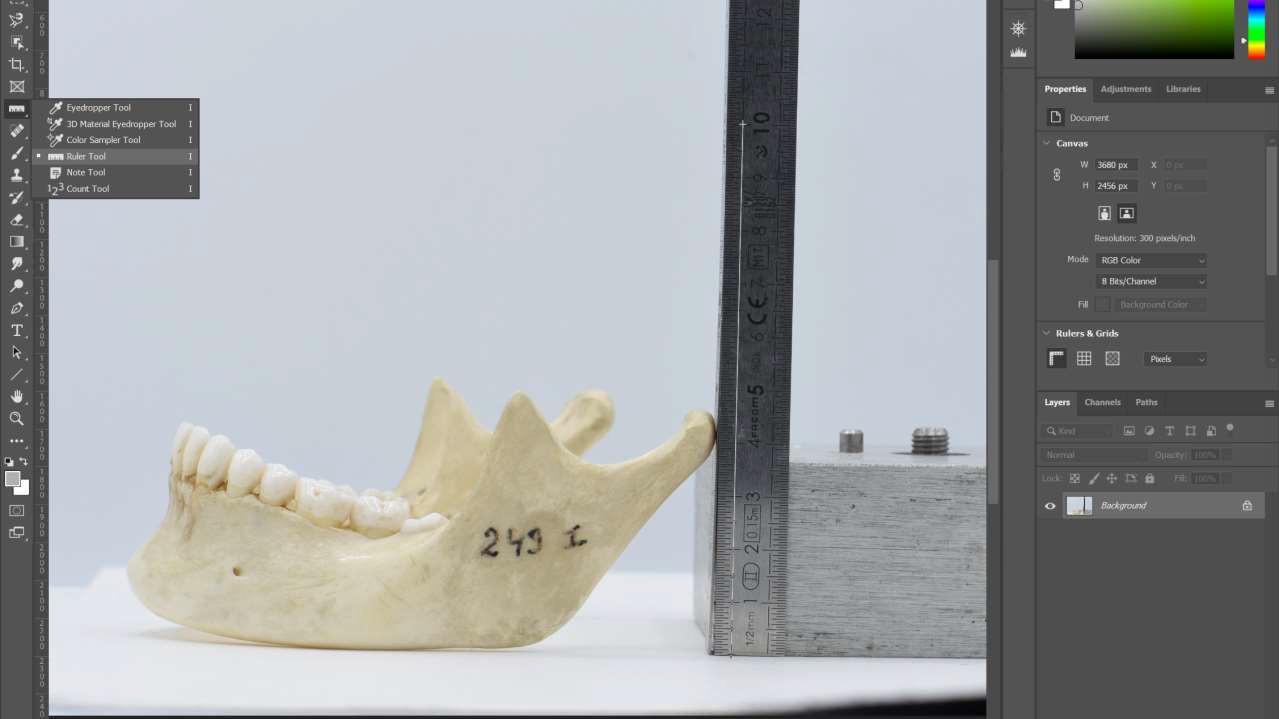


**Figure S1.** Select the ruler tool.


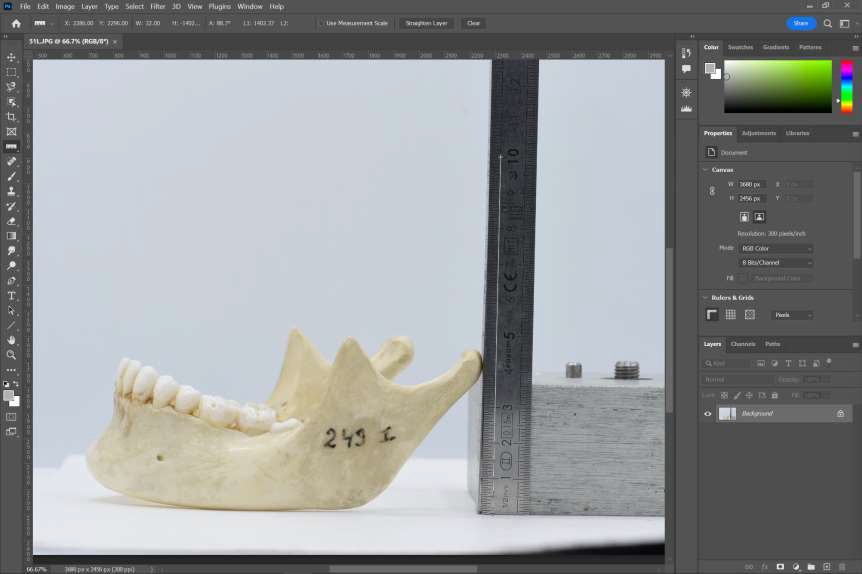


**Figure S2.** Measure 10cm on the ruler.


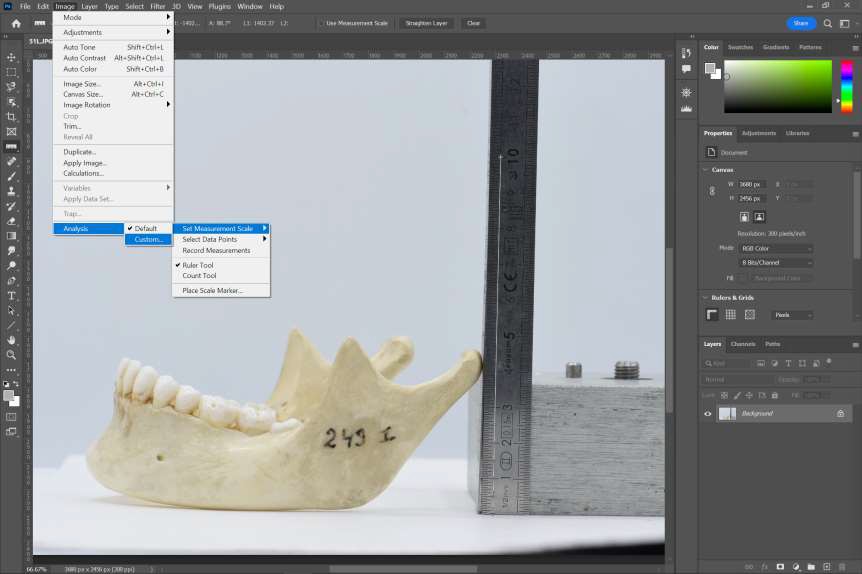


**Figure S3.** Go to ‘Set Measurement Scale’.


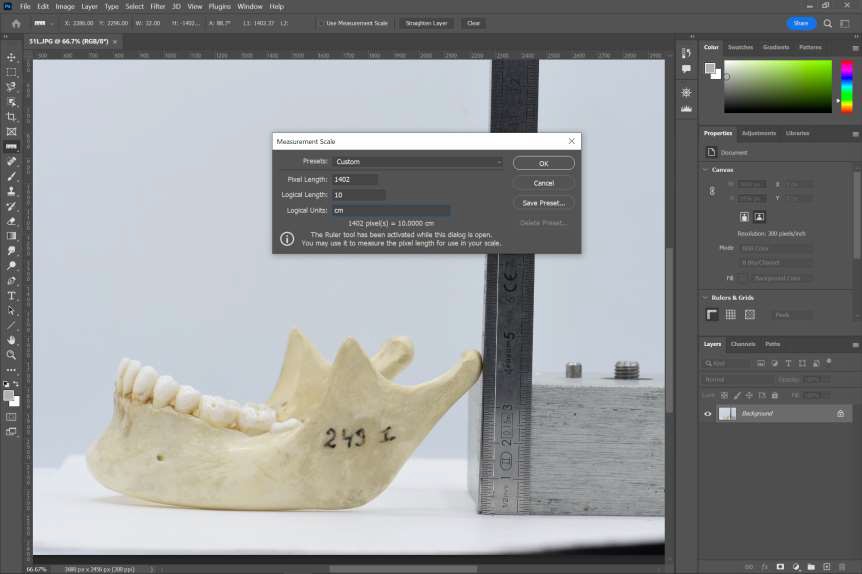


**Figure S4.** Enter the pixel length per 10cm.**
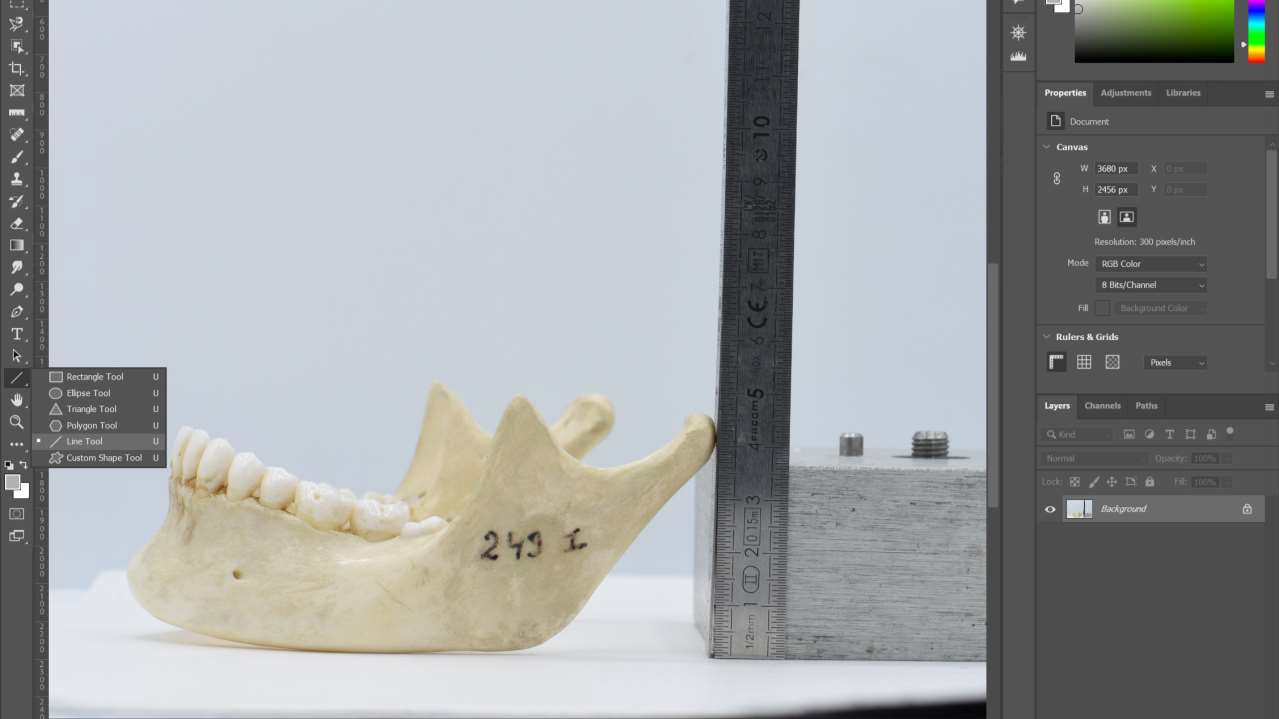
**

**Figure S5.** Select the line tool.

**
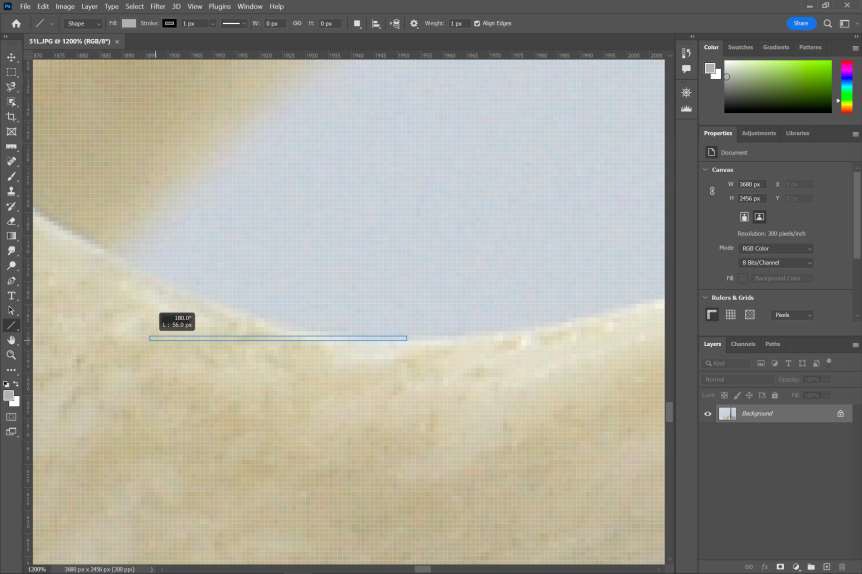
**

**Figure S6.** Select the middle of the lowest point of the incisura.

**
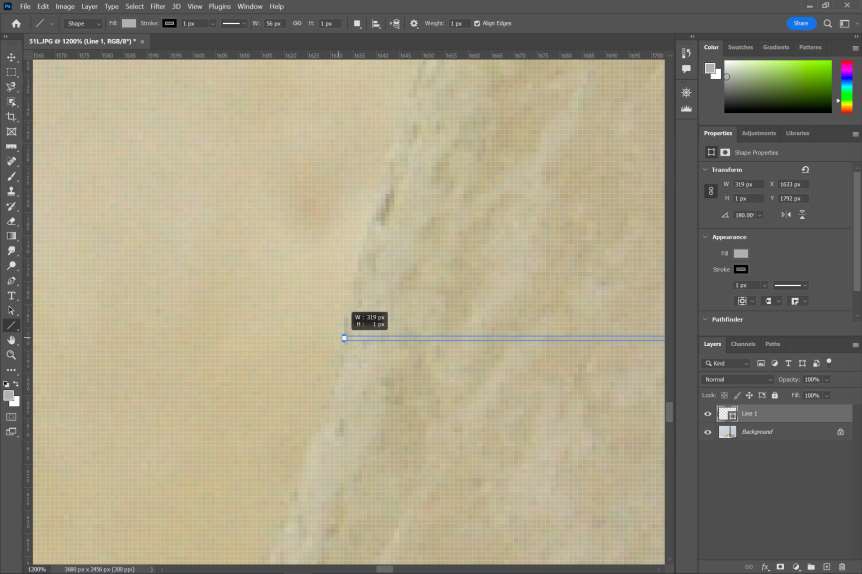
**

**Figure S7.** Extend the horizontal line up to the edge of the coronoid.


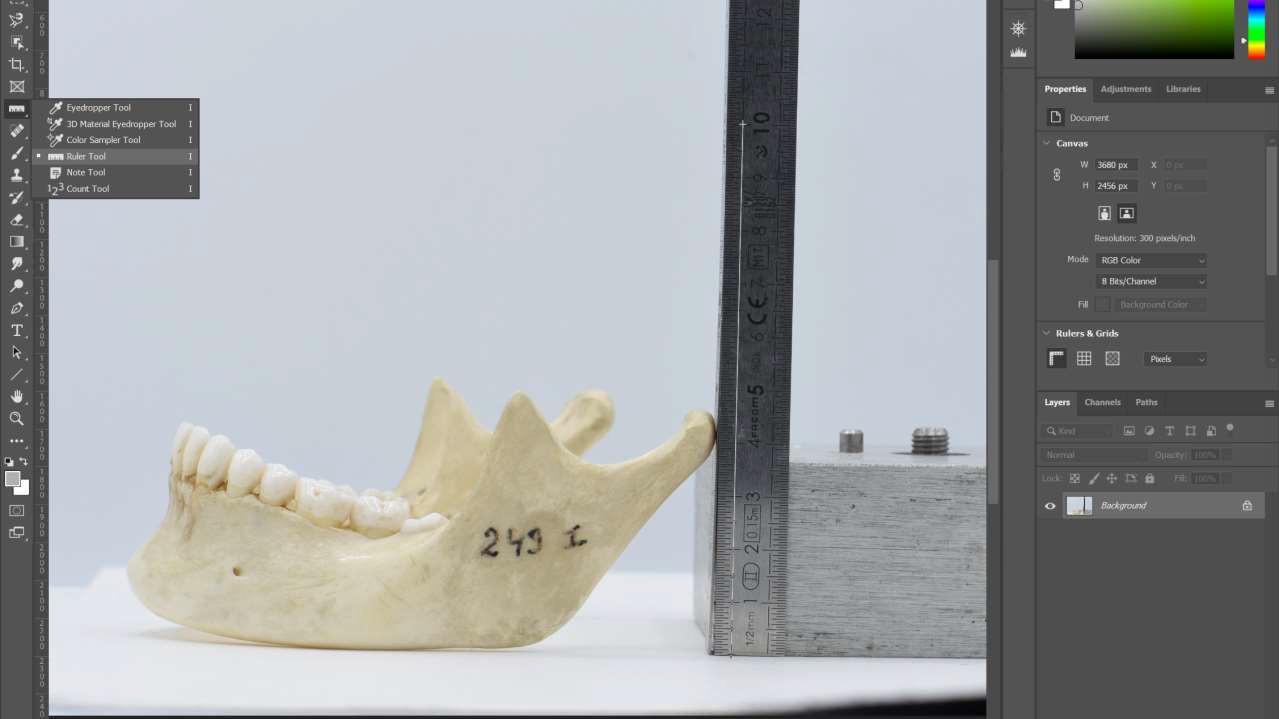


**Figure S8.** Select the ruler tool again.


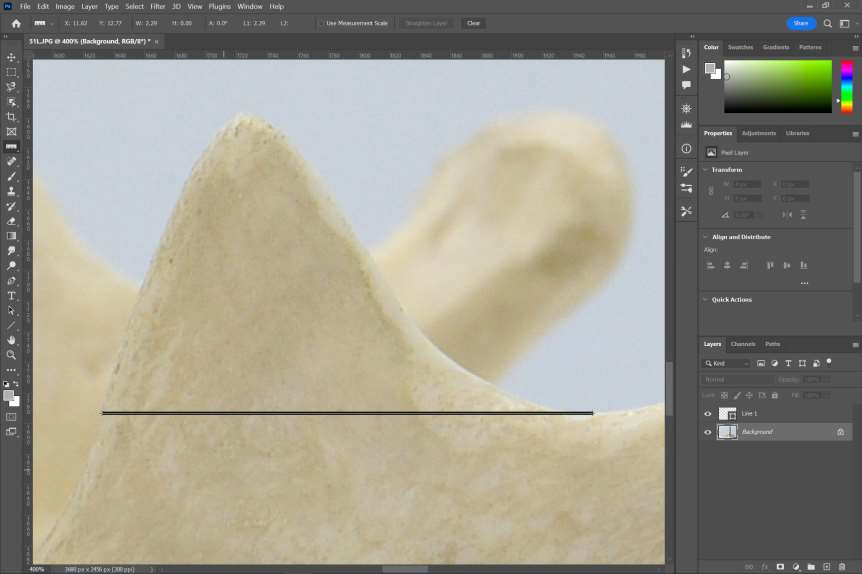


**Figure S9.** Measure width.


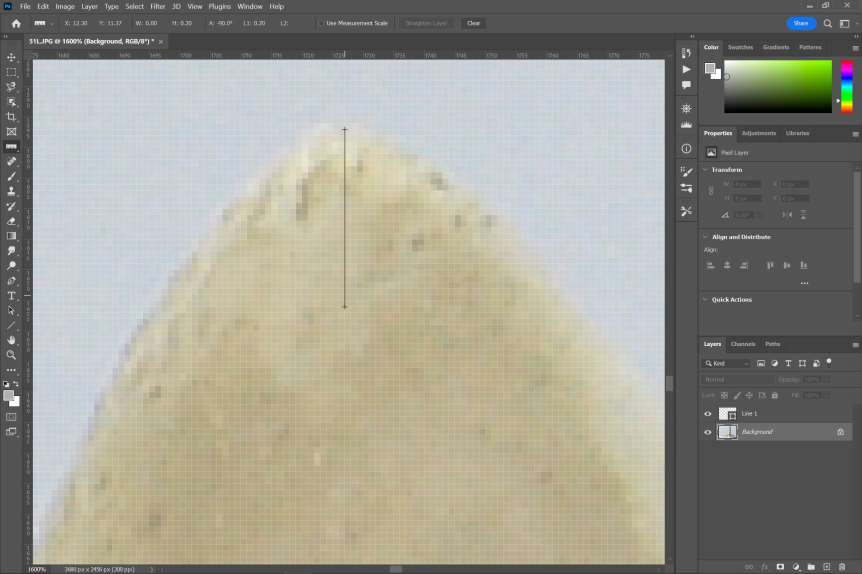


**Figure S10.** Select the middle of the highest point of the coronoid process.


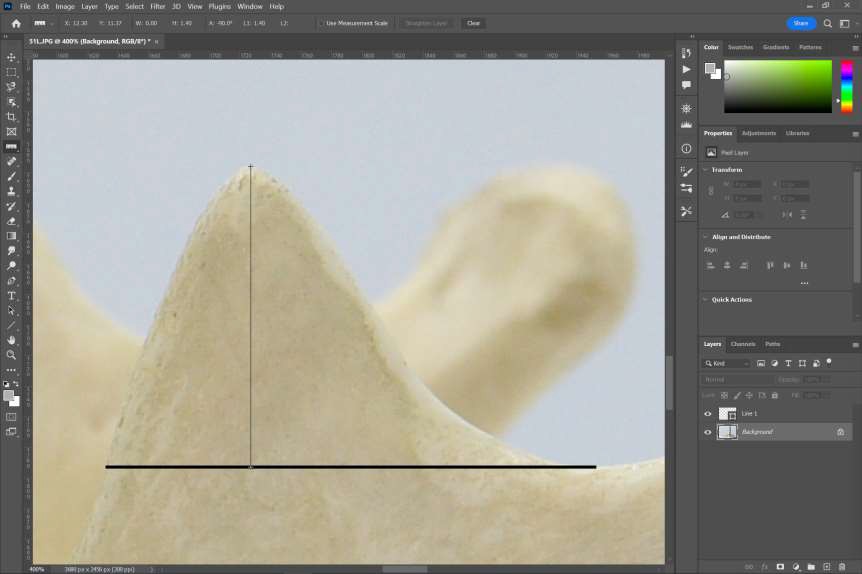


**Figure S11.** Measure height.


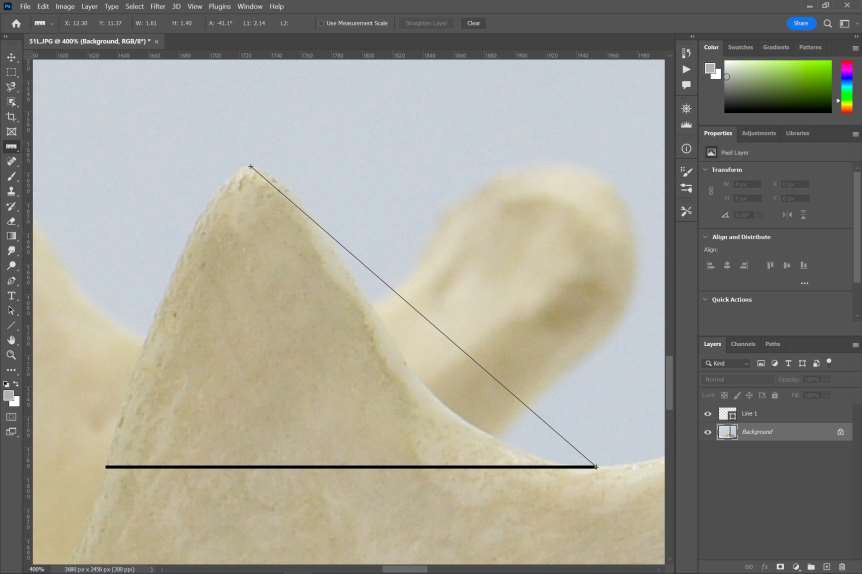


**Figure S12.** Measure length.


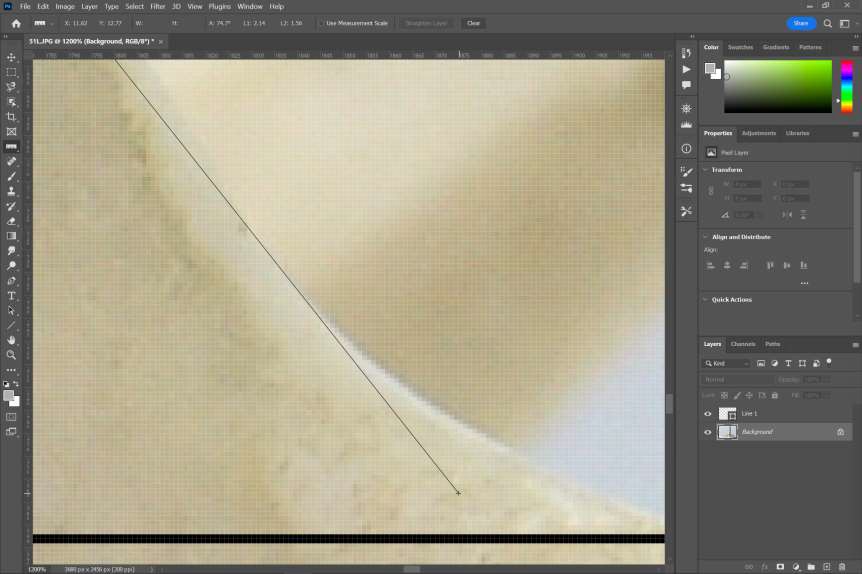


**Figure S13.** Select the tangent line alongside the coronoid process.


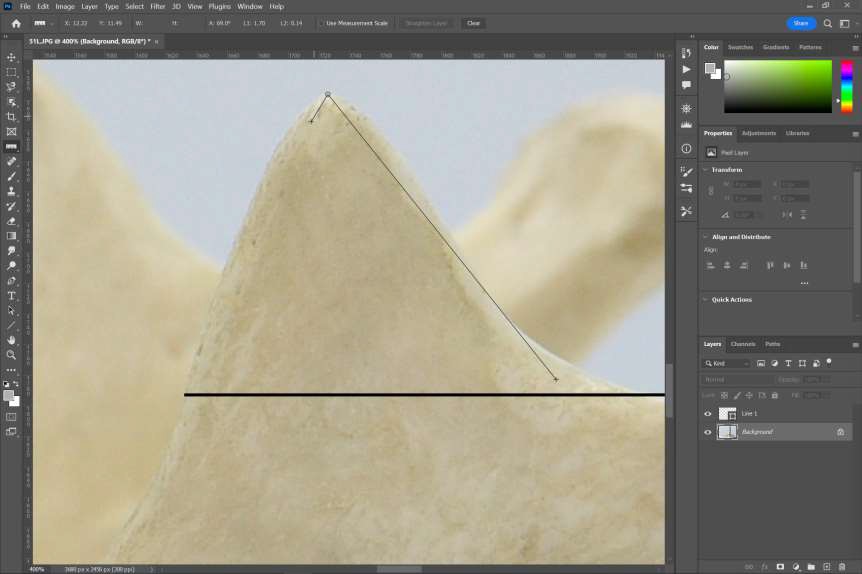


**Figure S14.** Use ‘alt’ at the highest point to measure an angle.


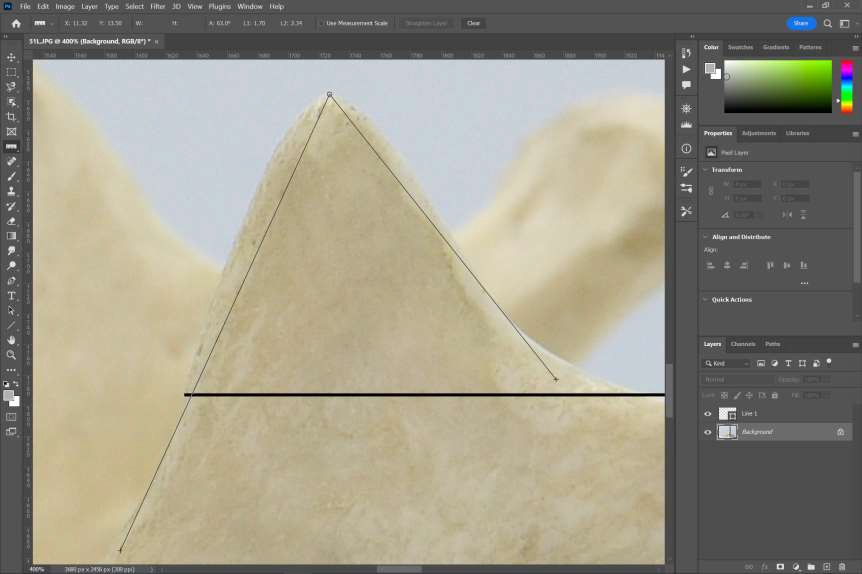


**Figure S15.** Select the other tangent line to measure angle X.

**
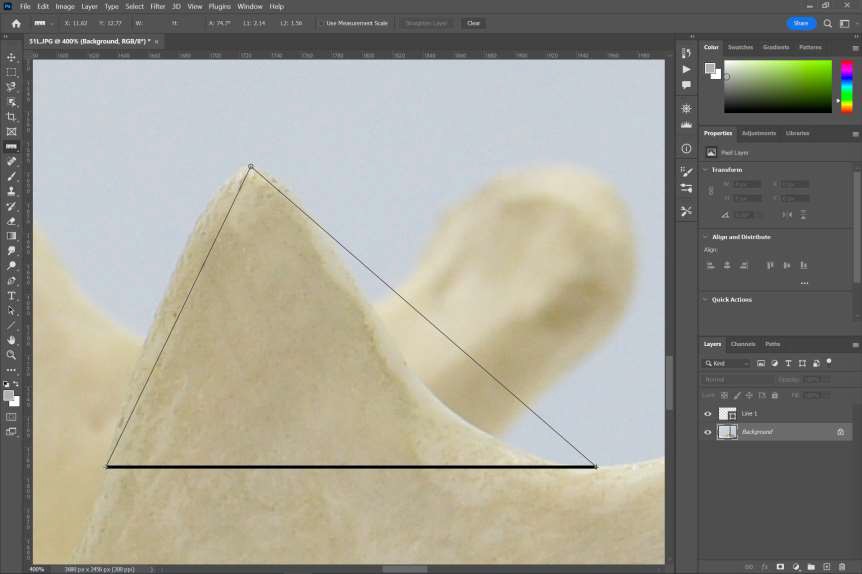
**

**Figure S16.** Measure angle Y.

**
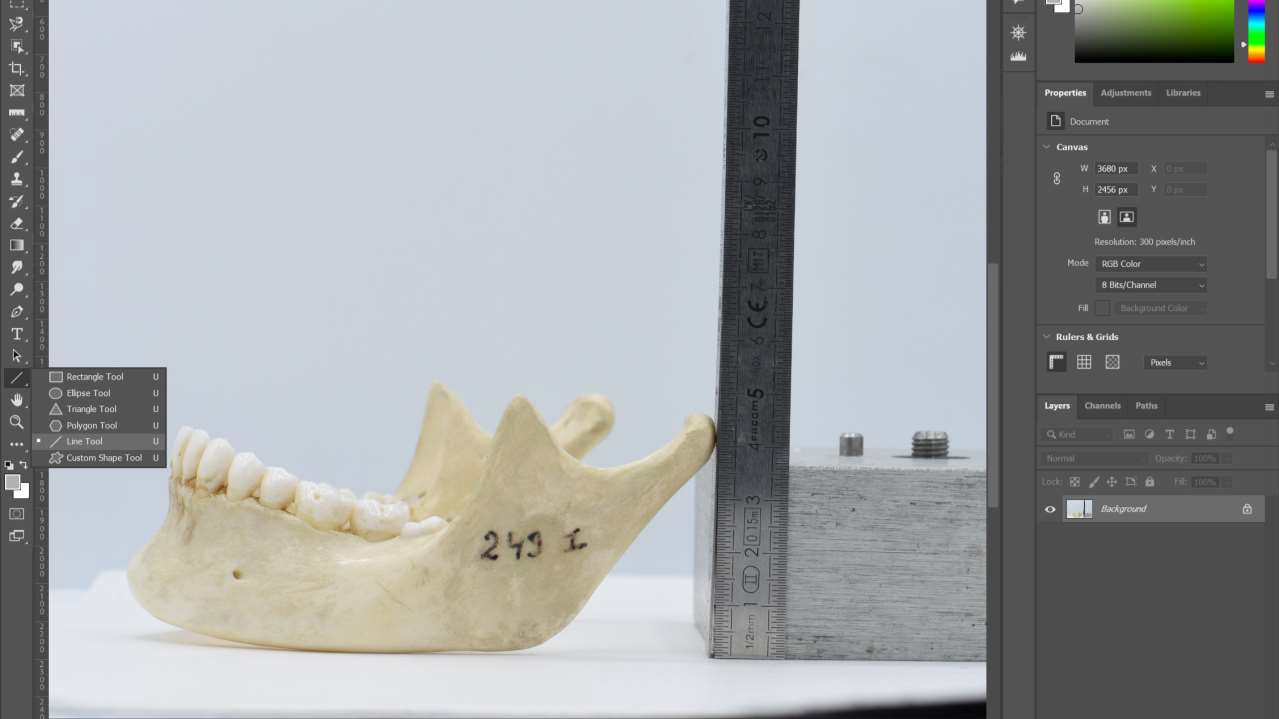
**

**Figure S17.** Select the line tool.

**
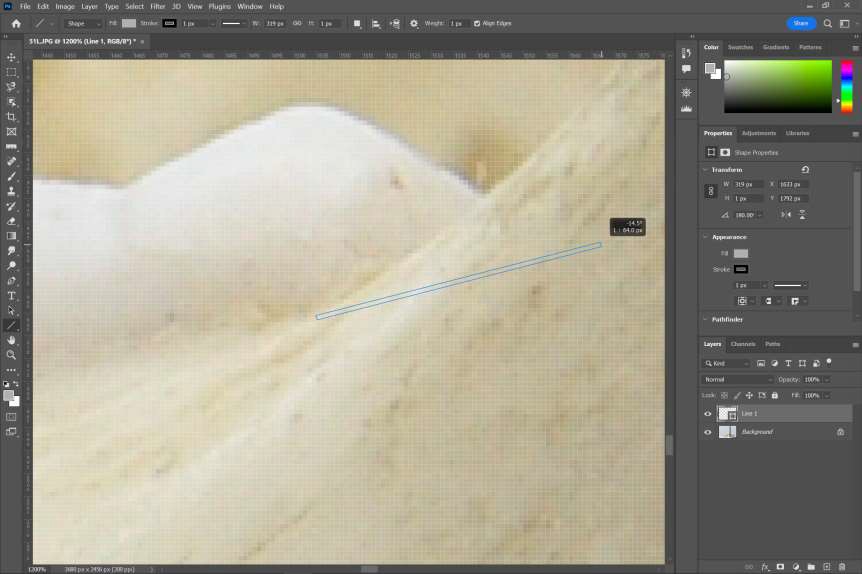
**

**Figure S18.** Select the intercept between coronoid and alveolar process and extend to lowest point of incisura.

**
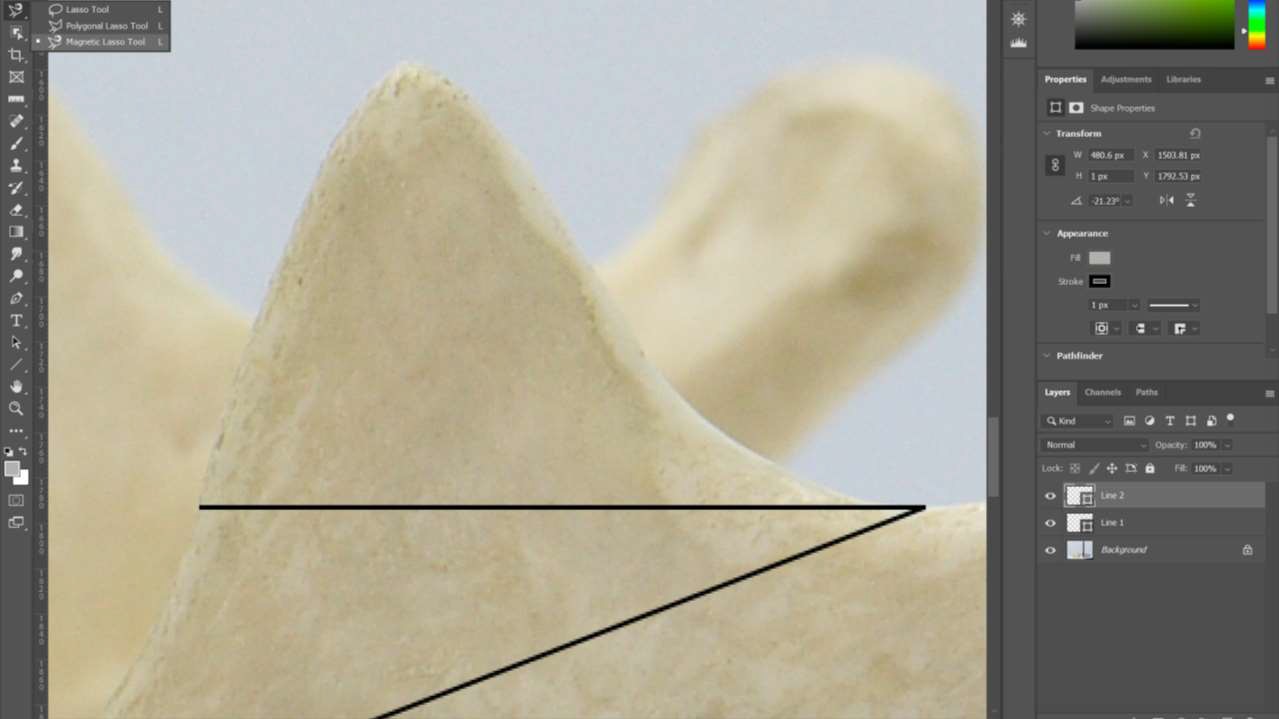
**

**Figure S19.** Select the magnetic lasso tool.

**
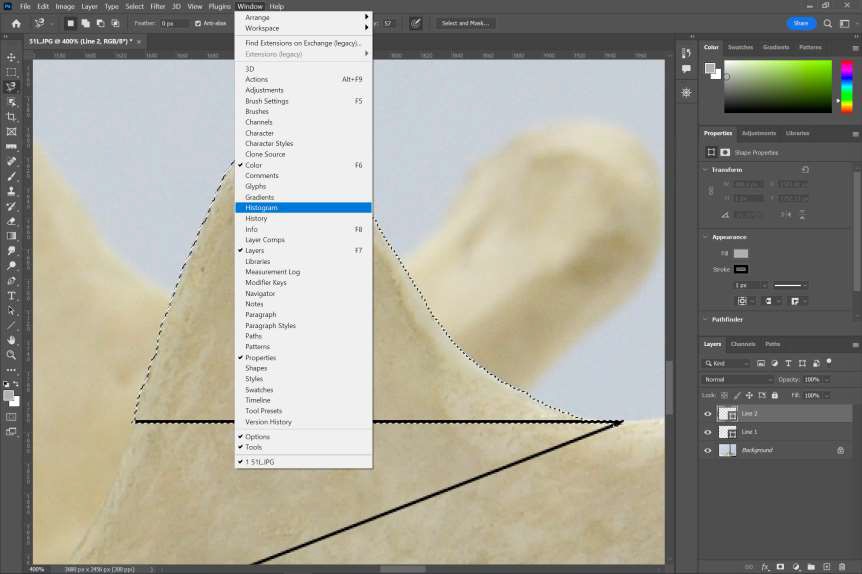
**

**Figure S20.** Enable the histogram.

**
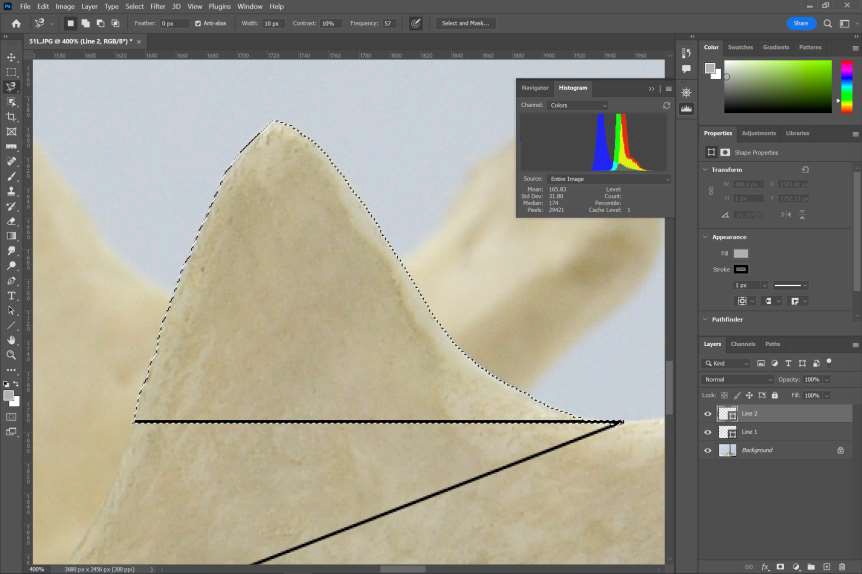
**

**Figure S21.** Measure surface area A with the magnetic lasso, pixels are seen in the histogram.

**
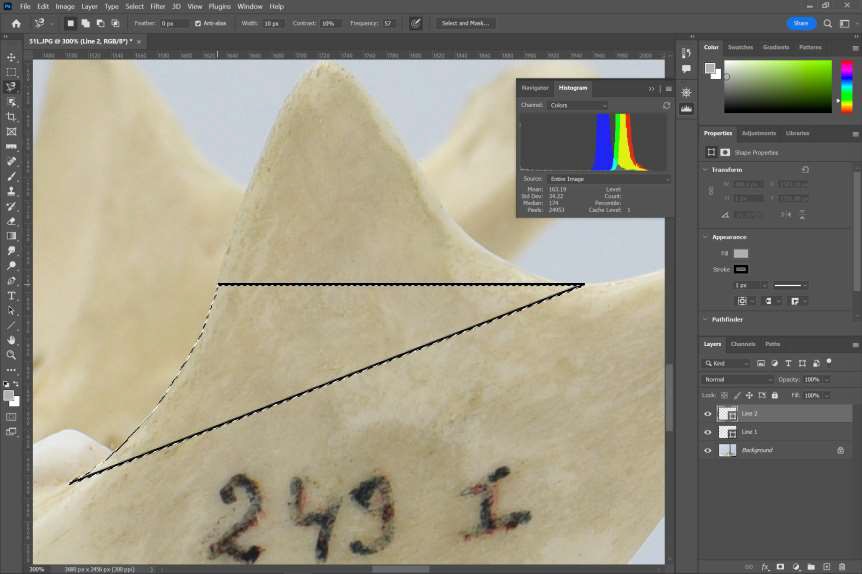
**

**Figure S22.** Measure surface area B.

**
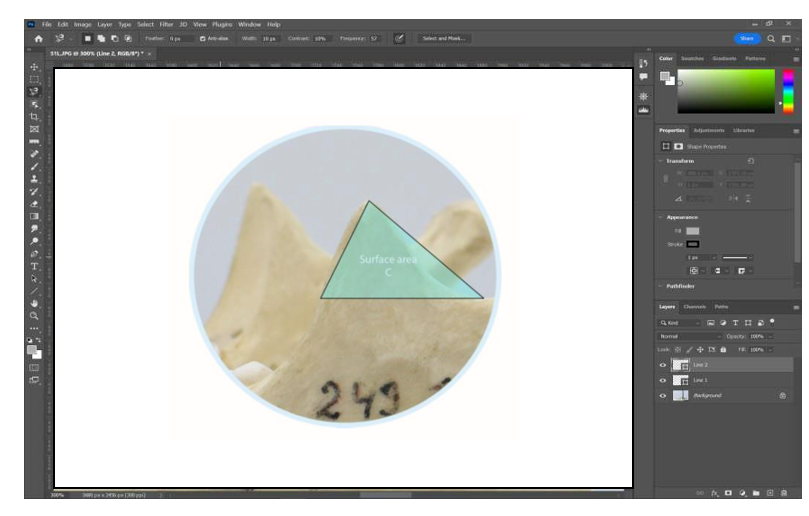
**

**Figure S23.** Measure surface area C.


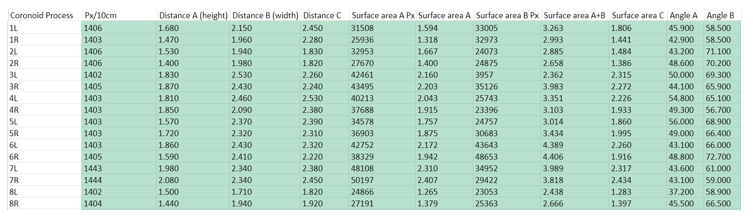


**Figure S24.** Fill in corresponding distances, surface areas and angulations in excel sheet.
